# Adherence to Enhanced Recovery After Surgery (ERAS) With Bellwether Surgical Procedures in Ethiopia: A Retrospective Study

**DOI:** 10.1002/wjs.12526

**Published:** 2025-03-20

**Authors:** Fitsum Kifle, Ermiyas Belay, Tewodros Kifleyohanes, Brook Demissie, Desta Galcha, Betelehem Mulye, Elise Presser, Ravi Oodit, Salome Maswime, Bruce Biccard

**Affiliations:** ^1^ Global Surgery Division Department of Surgery Faculty of Health Sciences University of Cape Town Observatory South Africa; ^2^ Network for Perioperative and Critical Care Debre Berhan University, Asrat Woldeyes Health science Campus Debre Berhan Ethiopia; ^3^ Department of Public Health College of Medicine and Health Science Wolkite University Wolkite Ethiopia; ^4^ Department of Surgery Debre Berhan University, Asrat Woldeyes Health science Campus Debre Berhan Ethiopia; ^5^ Department of Obstetrics and Gynaecology Alert Hospital Addis Ababa Ethiopia; ^6^ Department of Surgery College of Medicine and Health Sciences Arba Minch University Arba Minch Ethiopia; ^7^ Department of Quality and Health Management Information System Kidus Peteros Hospital Addis Ababa Ethiopia; ^8^ Department of Surgery Yale University New Haven Connecticut USA; ^9^ Department of Anaesthesia and Perioperative Medicine Groote Schuur Hospital University of Cape Town Cape Town South Africa

**Keywords:** Africa, bellwether, ERAS, postoperative, recovery

## Abstract

**Background:**

Enhanced recovery after surgery (ERAS) is a multimodal perioperative care approach that aims to improve patient outcomes by reducing physiological stress and promoting organ functional recovery. Implementing ERAS in low‐resource settings faces challenges due to limited infrastructure and resources. This study examined the adherence to five ERAS recommendations with Bellwether surgical procedures in Ethiopian surgical facilities.

**Method:**

A retrospective database review of the Ethiopian perioperative registry was conducted. A total of 555 patients were included in this study. Data extraction included patient demographics, American Society of Anesthesiologists' Physical Status classification, surgical variables, postoperative hospital length of stay (LOS), and ERAS guidelines components. The primary outcome was adherence to five ERAS guidelines recommendations (early mobilization, feeding initiation, postoperative nausea and vomiting prophylaxis, early catheter removal, and IV fluids discontinuation). The secondary outcomes included: (i) the association between adherence to ERAS guidelines and LOS and (ii) a total unduplicated reach and frequency analysis to determine the two recommendations with the most impact on decreasing LOS for future implementation in low‐resource environments.

**Results:**

A total of 555 patients were included across the three surgical categories: CS (274, 49.4%), OBF (126, 22.7%), and laparotomy (155, 27.9%). The primary outcome showed that the overall adherence was 1810 (65.2%) of the total number of the five ERAS guidelines recommendations in the cohort (2275 recommendations). The secondary outcomes showed that adherence to all five ERAS recommendations reduced LOS by 128 h compared to nonadherence to any ERAS elements. Adherence to early mobilization, early removal of urinary catheters, and early feeding each have shown consistent reductions in LOS across all Bellwether surgical procedures.

**Conclusion:**

The implementation of a limited set of ERAS recommendations in low‐resource environments has the potential to decrease LOS by approximately 5 days for Bellwether surgical procedures.

## Graphical abstract

Enhanced recovery after surgery (ERAS) is a multimodal perioperative care approach that aims to improve patient outcomes by reducing physiological stress and promoting organ functional recovery. Implementing ERAS in low‐resource settings faces challenges due to limited infrastructure and resources. This study examined the adherence to five ERAS recommendations with Bellwether surgical procedures in Ethiopian surgical facilities. A retrospective database review of the Ethiopian perioperative registry was conducted. A total of 555 patients were included in this study.

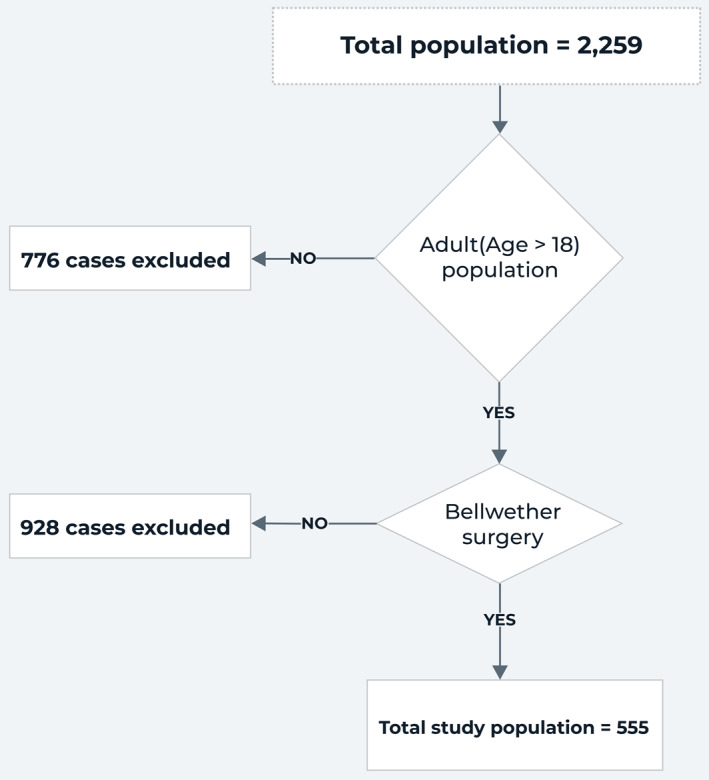

## 1 Background

Enhanced recovery after surgery (ERAS) is a multimodal multidisciplinary approach to perioperative care that aims to improve patient outcomes by reducing physiological stress and promoting organ function recovery by optimizing preoperative preparation, intraoperative care, and postoperative management [1]. ERAS guidelines have been widely implemented in high‐income countries and have been shown to reduce complications, LOS, and costs, while maintaining or improving clinical outcomes [2, 3, 4]. However, applying ERAS guidelines in low‐resource settings is not without challenges, requiring local adaptation and innovation due to the significant infrastructure, staffing, and financing challenges faced by patients and healthcare systems [1, 5].

Despite these complexities, there are emerging examples of successful ERAS programs in low‐resource settings that leverage existing resources, engage local stakeholders, and prioritize patient‐centered care [6, 7]. For example, a study in rural Uganda found that implementing ERAS guidelines for cesarean section (CS) surgery reduced pain, headaches, and hospital LOS [8], whereas a study in India demonstrated that an ERAS program for colorectal surgery was feasible and effective in improving postoperative recovery [9].

Ethiopia, a low‐income country located in East Africa and the second most populous country in the region, has made significant progress in improving its surgical capacity [10]. Ethiopia now has national perioperative guidelines with integrated ERAS recommendations [11]. However, the implementation of these guidelines and their impact on patient outcomes remain unknown due to the lack of routine patient‐level data and systematic auditing. To address this knowledge gap, the Network for Perioperative and Critical Care (N4PCc) [12] established a perioperative registry where surgical information is captured prospectively throughout the perioperative journey, supporting the establishment of the Ethiopian National Perioperative Quality Improvement Network (NaPQN) [13]. Trained personnel, ranging from qualified medical practitioners to diploma‐level nurses, gather these data depending on the location and capacity of the hospital to ensure its accuracy and reliability.

Using this registry, this study aimed to evaluate: (1) the level of adherence to ERAS guidelines in Ethiopian surgical facilities for Bellwether surgical procedures; (2) the correlation between adherence to ERAS guidelines and patient outcomes; and (3) the differences in postoperative adherence to ERAS guidelines between the different Bellwether surgical procedures. Bellwether procedures were defined as an open laparotomy, cesarean section (CS), or treatment of an open bone fracture (OBF) [14]. The findings of this study will provide valuable insights into the implementation of ERAS guidelines in Ethiopia and inform future strategies for improving ERAS practices.

## 2 Method

### 2.1 Study Type and Outcomes

This was a retrospective study of adherence to ERAS guidelines recommendations in Ethiopian hospitals. Eligible patients were adults (aged ≥ 18 years) patients undergoing Bellwether procedures in facilities with ERAS guidelines for these surgeries [13]. The primary outcome was adherence to five postoperative ERAS guidelines recommendations (early mobilization, initiation of feeding, postoperative nausea and vomiting [PONV] prophylaxis, early urinary catheter removal, and early discontinuation of intravenous fluids). These ERAS elements were chosen due to their availability for daily measurement in the national database and alignment with LMIC guidelines as part of the postoperative ERAS recommendations. The secondary outcomes included: (i) the association between adherence to ERAS guidelines recommendations and LOS and (ii) a total unduplicated reach and frequency (TURF) analysis to determine which two of the five ERAS guidelines recommendations are commonly practiced together and show the most impact on decreasing LOS with future implementation in low‐resource environments. Adherence was defined as the number of ERAS guidelines recommendations that were completed of the total number of recommendations within the patient cohort (5 ERAS guidelines recommendations per patient within the cohort).

### 2.2 Sampling Method

**FIGURE 1:**
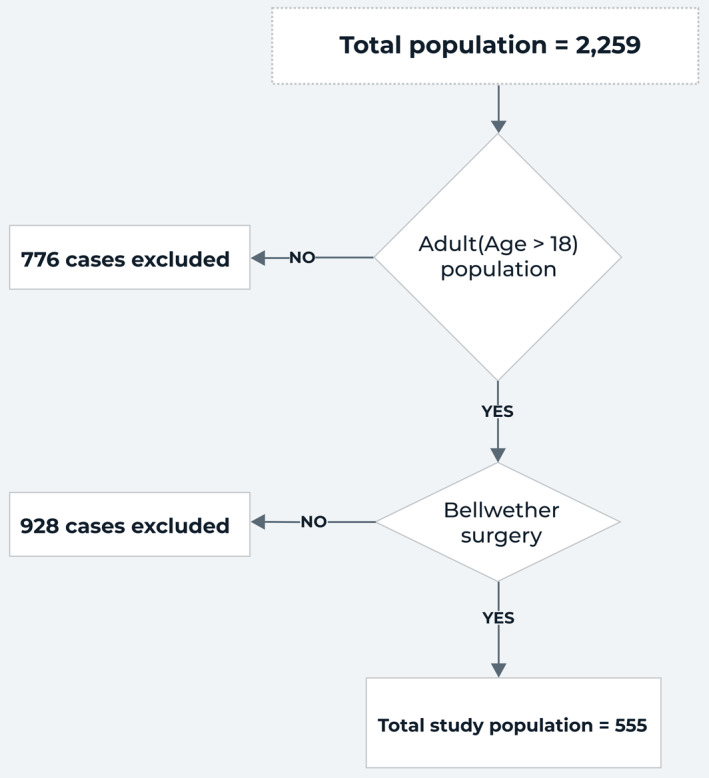
Summary of the sampling procedures used.

The sample was drawn from patient records in the NaPQN perioperative registry of seven surgical facilities and the level of hospitals can be found in the Supporting Information S1: Table S1. To ensure completeness of current and accurate data, we restricted our sample to patients who underwent surgical intervention from August 28 to October 30, 2023 (inclusive) and underwent Bellwether surgical procedures. We excluded records before August 28, 2023, as this was when the registry tool was updated to include more specific data points related to ERAS guidelines recommendations. This resulted in a final population of 2259. Figure 1 further shows the recruitment process.

The sample size determination was conducted using the single population proportion formula available in the OpenEpi open‐source statistical software [15]. Assuming a 50% estimated proportion of the outcome of interest, a margin of error of 5%, and a 95% confidence level, the required sample size was calculated to be 348 participants. To account for potential nonresponse, a 5% adjustment was applied, confirming 348 as the minimum sample size necessary for robust analysis. However, recognizing the value of maximizing data utility and enhancing the generalizability of the study’s conclusions, we included all 555 eligible patient records from the pooled data to allow for a more representative analysis and strengthen the external validity of the findings.

### 2.3 Data Extraction and Analysis

Data extraction included patient demographics, American Society of Anesthesiologists (ASA), physical status classification, urgency of surgery, and intraoperative variables, including surgery type. Outcome variables included hospital LOS and ERAS guidelines recommendation components. The guideline’s recommendations were time to mobilization, time to initiation of feeding, postoperative nausea and vomiting (PONV) prophylaxis, time to urinary catheter removal, and time of discontinuation of intravenous fluids. For missing value management, we examined the magnitude and patterns of the variables that were central to the analysis. The pattern of missingness does not dictate systematic appearance and there is roughly randomized missingness across the data.

In order to identify missing patterns in our dataset, we used visual and statistical (Little’s test) methods. We used the multiple imputation (MI) technique to manage the missing data. Most of the variables were categorical, and our study was exploratory. Besides the missing data pattern, MI has been found to produce better results under these conditions [16]. Although multiple imputations perform well under the assumption that the missing data are random, it does not necessitate the specific pattern to be random [17].

Descriptive statistics were employed to summarize and present the data through tables and statistical summaries. The chi‐squared test was used to evaluate adherence to ERAS protocols across different Bellwether surgical procedures. The *t*‐test was utilized to compare LOS between adherent and nonadherent groups. A one‐way analysis of variance (ANOVA) post hoc analyses, that is Tukey's honestly significant difference (HSD) test, were performed to investigate significant differences in the mean hospital length of stay among varying levels of ERAS adherence within these surgeries. Regression analysis was also conducted to identify potential predictors of LOS associated with ERAS practices. A significance level of 5% was applied to all statistical tests. Total unduplicated reach and frequency (TURF) analysis was also utilized to evaluate the frequency of ERAS adherence frequency in the study population and identify the most common combinations when all five ERAS practices could not be implemented at once.

One limitation of TURF analysis is that it assigns equal weight to all components based solely on frequency, which may not reflect the clinical importance of each component. To address this, we used TURF analysis purely as a descriptive tool to understand current adherence patterns rather than to prioritize specific practices over others. Additionally, to minimize bias from missing data, we employed multiple imputation methods, ensuring a more accurate representation of the dataset. All data were analyzed using the R statistical software version 4.4.2.

### 2.4 Operational Definitions

The national perioperative guidelines suggest early feeding and mobilization, but for a more complete assessment, we utilized five of the ERAS LMIC perioperative recommendations [5], as the database permits this definition. These were defined as follows:

1. Early mobilization: walking within 24 h postsurgery.
2. Early urinary catheter removal: removal within 24 h postsurgery.
3. Early discontinuation of intravenous fluid (IV): discontinuation of IV fluids within 24 h postsurgery.
4. Postoperative nausea and vomiting prophylaxis (PONV): administration of antiemetic medications before surgery to prevent postoperative nausea and vomiting.
5. Early initiation of feeding: commencing oral intake of fluids immediately after surgery, with solids introduced after 4 h postsurgery.

## 3 Results

### 3.1 Sociodemographics of the Patients in the Study

Figure 1 shows the patient recruitment. The characteristics of the participating hospitals are shown in Supporting Information S1. This study found over 91% completeness in the variables for the analysis, with most having missingness below 5%. All statistical analyses used complete case analysis, except for the logistic regression in machine learning, where multiple imputations are applied via the multiple imputation by chained equations (MICE) package in R after observing random missing patterns in a heatmap. The magnitude and pattern of missing data are further shown in the Supporting Information S1: Figure S1.

Patient characteristics are presented in Table 1. A total of 555 patients were included in this study: 308 scheduled cases (55%) and 247 nonscheduled (emergency) cases 247 (45%) undergoing Bellwether surgical procedures: cesarean sections (274, 49.4%), OBF (126, 22.7%), and laparotomies (155, 27.9%).

**TABLE 1:** Patient demographics and characteristics.

			Type of surgical category		
Variable	*N*	Overall *N* = 555^a^	OBF *N* = 126^a^	CS *N* = 274^a^	Laparotomy *N* = 155^a^
Sex	550				
Male		150 (27%)	72 (60%)	0 (0%)	78 (50%)
Female		400 (73%)	49 (40%)	274 (100%)	77 (50%)
Age	543				
18–29.9		248 (46%)	39 (31%)	159 (61%)	50 (32%)
30–50.9		216 (40%)	56 (44%)	103 (39%)	57 (37%)
51–65		46 (8.5%)	18 (14%)	0 (0%)	28 (18%)
> 65		33 (6.1%)	13 (10%)	0 (0%)	20 (13%)
Marital status	553				
Married		462 (84%)	86 (69%)	256 (94%)	120 (77%)
Single		68 (12%)	26 (21%)	10 (3.7%)	32 (21%)
Other		23 (4.2%)	13 (10%)	7 (2.6%)	3 (1.9%)
Educational status	546				
Illiterate		77 (14%)	21 (17%)	20 (7.4%)	36 (24%)
Elementary		124 (23%)	24 (20%)	57 (21%)	43 (28%)
Highschool		123 (23%)	20 (16%)	71 (26%)	32 (21%)
College and above		113 (21%)	19 (16%)	60 (22%)	34 (22%)
Other		109 (20%)	38 (31%)	64 (24%)	7 (4.6%)
Occupation	551				
Gov’t employed		48 (8.7%)	6 (4.8%)	31 (11%)	11 (7.1%)
Private work		151 (27%)	50 (40%)	74 (27%)	27 (18%)
Student		36 (6.5%)	11 (8.8%)	10 (3.7%)	15 (9.7%)
Housewife		169 (31%)	21 (17%)	110 (40%)	38 (25%)
Other		147 (27%)	37 (30%)	47 (17%)	63 (41%)
Primary source of payment	549				
Out of pocket		158 (29%)	77 (62%)	26 (9.6%)	55 (36%)
Tena medhin/health insurance		156 (28%)	40 (32%)	30 (11%)	86 (56%)
Sponsored by NGO		45 (8.2%)	3 (2.4%)	42 (15%)	0 (0%)
Free service		32 (5.8%)	2 (1.6%)	26 (9.6%)	4 (2.6%)
Other		158 (29%)	3 (2.4%)	147 (54%)	8 (5.2%)
Admission status	549				
Direct admission		268 (49%)	68 (54%)	135 (50%)	65 (42%)
Referral		281 (51%)	57 (46%)	134 (50%)	90 (58%)
Surgical urgency	555				
Scheduled		247 (45%)	103 (82%)	72 (26%)	72 (46%)
Nonscheduled		308 (55%)	23 (18%)	202 (74%)	83 (54%)
ASA classification	515				
I		150 (29%)	48 (42.8%)	51 (19%)	51 (37%)
II		338 (65.6%)	61 (54.5%)	214 (81%)	63 (45.7%)
≥ III		27 (5.2%)	3 (2.7%)	0 (0%)	24 (17.3%).

*Note:* “Scheduled” includes both “elective” and “expedited” cases as defined by the National Confidential Enquiry into Patient Outcome and Death (NCEPOD) classification (The NCEPOD Classification of Intervention). Available at: https://www.ncepod.org.uk/classification.html (Accessed: 19 January 2025).

Abbreviations: ASA, American Society of Anesthesiologists; CS, cesarian section; Gov’t, government; and OBF, open bone fracture.

^a^ *n/N* (%).

Males constituted 150 (27%) of the total sample, predominantly in the laparotomy 78 (52%) and OBF 72 (48%) categories, whereas females represented 400 (73%), mainly in the CS category 274 (68.5%). In the cases where gender was incorrectly recorded as male for the CS category, 15 (5.5%) were cross‐checked and manually corrected to female. All patients fell into the ASA I and II groups except for 27 (5.2%) in ASA III, mainly within the laparotomy group (24/27; 88%). The largest age group was 18–29.9 years (248/555; 45%), followed by 30–50.9 years (217/555; 39%). The majority of patients were married (462/553; 84%), and those with elementary and high school education comprised the largest educational subgroup 23% each. Private work was the main occupation (151/551; 27%). Payment for medical services was primarily out‐of‐pocket (158/549; 29%), with government insurance (156/549; 28%) and minimal government‐free service 32 (5.8%), mainly for the CS group 26 (82%). The adherence to the ERAS guidelines and recommendations is shown in Table 2, and the association with LOS is shown in Table 3.

**TABLE 2:** ERAS practice across 555 Bellwether procedures in seven hospitals in Ethiopia **(**Pearson’s chi‐squared test).

			Type of surgical category			
Variable	*N*	Overall	OBF	CS	Laparotomy	*p*‐value
		*N* = 555^a^	*N* = 126^a^	*N* = 274^a^	*N* = 155^a^	
Early fluid balance	540	391/540 (72%)	62/114 (54%)	227/271 (84%)	102/155 (66%)	< 0.001
Early catheter removal	555	423/555 (76%)	82/126 (65%)	238/274 (87%)	103/155 (66%)	< 0.001
Early PONV	555	379/555 (68%)	67/126 (53%)	200/274 (73%)	112/155 (72%)	< 0.001
Early feeding	555	308/555 (55%)	75/126 (60%)	184/274 (67%)	49/155 (32%)	< 0.001
Early mobilization	541	309/541 (57%)	28/114 (25%)	224/273 (82%)	57/154 (37%)	< 0.001

Abbreviations: OBF, open bone fracture and PONV, postoperative nausea and vomiting prophylaxis.

^a^ *n/N* (%).

**TABLE 3:** T‐test summary table: mean hospital length of stays (LOS) across the five ERAS element**s**.

ERAS element		Mean LOS among the types of procedure			Mean difference in hours (95% CI)	T‐value	*p*‐value
		OBF	CS	Laparotomy			
		*N* = 126	*N* = 274	*N* = 155			
Early mobility (*n* = 555)	Yes	148.91	94.87	134.91	86.97 (38.33, 135.61)	3.54	< 0.001
	No	274.97	228.18	134.50			
Early catheter removal (*n* = 541)	Yes	125.50	88.14	123.17	80.87 (50.19, 111.56)	5.19	< 0.001
	No	193.33	224.70	141.18			
Early fluid balance (*n* = 540)	Yes	175.25	116.91	108.97	35.1 (3.35, 66.85)	2.18	0.03
	No	189.60	88.14	183.22			
Early PONV prophylaxis (*n* = 555)	Yes	187.15	121.61	133.38	−8.46 (34.61, 17.69)	−0.64	0.525
	No	181.24	88.47	138.17			
Early feeding (*n* = 555)	Yes	165.54	104.34	130.93	23.85 (−4.37, 52.08)	1.66	0.097
	No	220.11	127.88	136.63			

Abbreviations: CI, confidence interval; CS, caesarian section; OBF, open bone fracture; and PONV, postoperative nausea vomiting.

### 3.2 Early Mobilization

Most patients (309 [57%]) mobilized within 24 h postoperatively as shown in Table 2. Early mobilization was the most prevalent among patients who underwent CS (224/309, 72.5%) compared to (28/309, 9.1%) in OBF cases and (57/309, 18.4%) among emergency laparotomy cases (*p* < 0.001). Patients who mobilized within 24 h postoperatively had a shorter average hospital LOS, with a difference of 87 h compared to those who did not ambulate early (*t* = 3.54 and *p*‐value < 0.001), as shown in Table 3.

### 3.3 Postoperative Urinary Catheter

Postoperative urinary catheter removal within the first 24 h was observed in 76% (423/555) of cases, with the highest rate in the CS group at 87% (238/274). Conversely, 24% (132/555) of patients did not have their catheters removed within 24 h, with the OBF group comprising a significant portion, 35% (44/126) of these cases (Table 2). Among those whose catheters were removed within 24 h, the average hospital stays were 88.14 h for CS, 125.50 h for OBF, and 123.17 h for laparotomy cases. In contrast, patients with catheters retained beyond 24 h experienced longer hospital stays of 224.70, 193.33, and 141.18 h, respectively. The duration of catheter placement is significantly associated with a mean difference of 80.87 h (*p* < 0.001) as shown in Table 3.

### 3.4 Fluid Balance

In 391/540 (72%) of patients, primarily in the CS group (227/271, 84%), intravenous fluids were stopped within 24 h as shown in Table 2. Among those who had their IV fluids discontinued within 24 h postsurgery, hospital stays were notably shorter compared to patients who received fluids for more than 24 h. This trend was also observed in other groups, with 54% (62/114) of OBF patients and 66% (102/155) of laparotomy patients having IV fluids stopped within 24 h. The average reduction in hospital stay for early IV fluid discontinuation was 35.1 h (*p* = 0.03) as shown in Table 3.

### 3.5 PONV Prophylaxis

Postoperative nausea and vomiting (PONV) prophylaxis was administered to 68% of patients, with adherence rates across the three groups shown in Table 3. There was no significant difference in LOS between patients who did not receive PONV prophylaxis (127.88 h) and those who did (136.33 h), *p* = 0.525.

### 3.6 Early Feeding

Of the patients, 308/555 (55%) received early oral intake. Early feeding was initiated in 75/308 (24.4%) OBF, 184/308 (59.7%) in CS cases, and 49/308 (15.9%) in laparotomy patients. The average hospital stay was shorter for the early‐fed group compared to those fed later, except for the OBF group, as shown in Table 3.

### 3.7 Level of Adherence to the Five ERAS Elements

For the primary outcome, adherence to the five ERAS practices was observed in 65.2% (1810) out of a total of 2775 possible instances (five recommended practices for each 555 patients) as can be observed from Table 4. Moreover, the TURF analysis demonstrated that the combination of “Early IV fluid discontinuation” and “PONV prophylaxis” resulted in an unduplicated unique reach of 514 cases (92.6%). Notably, adherence to early urinary catheter removal and early mobilization showed the strongest association with reduced hospital LOS as detailed in Table 3.

**TABLE 4:** The proportion of recommended practices for five ERAS elements among 555 Bellwether procedure**s**.

Unique reach and frequency for each combination			
Combination	ERAS element	Individual frequency	Unique reach
1	Early fluid balance	391	514
	Early PONV prophylaxis	379	
2	Early fluid balance	391	491
	Early mobility	423	
3	Early mobility	423	479
	Early PONV prophylaxis	379	
4	Early fluid balance	391	478
	Early feeding	308	
5	Early PONV prophylaxis	379	469
	Early feeding	308	
6	Early PONV prophylaxis	379	467
	Early catheter	309	
7	Early mobility	423	450
	Early catheter	309	
8	Early mobility	423	448
	Early feeding	308	
9	Early fluid balance	391	438
	Early catheter	309	
10	Early feeding	308	413
	Early catheter	309	

*Note:* N.B: Unique reach represents how many times this specific set of practices (collectively or separately) impacted different cases without counting any single instance more than once. A higher unique reach suggests that the combination appeals to a larger audience, thereby maximizing coverage. By adding up each frequency and dividing it by the total number of unique cases reached, one can calculate the overall adherence.

Abbreviations: PONV: postoperative nausea vomiting.

### 3.8 Association Between ERAS Practice and LOS

A post hoc analysis was made to conduct an ANOVA test to compare hospital LOS across the six categories of adherence to recommendations (none to adherence to all five components). Adherence to all five components reduced LOS by 128 h compared to none (*p* = 0.018). Similarly, adherence to one, two, or three components also resulted in shorter hospital LOS, with reductions ranging from 48 to 65 h, as can be seen in Table 5.

**TABLE 5:** Post hoc test showing the differences in mean hospital length of stay (LOS) among a range of ERAS practices in 555 bellwether surgerie**s**.

Comparison (between the number of ERAS elements practiced)	Difference LOS (hours)	Lower 95% CI	Upper 95% CI	*p*‐value
1–0	−68.412	−192.572	55.748	0.615
2–0	−36.201	−155.844	83.442	0.954
3–0	−47.761	−162.605	67.084	0.842
4–0	−112.318	−221.989	−2.648	0.041
5–0	−128.273	−243.014	−13.532	0.018
2–1	32.211	−55.583	120.005	0.901
3–1	20.651	−60.482	101.784	0.978
4–1	−43.907	−117.533	29.720	0.528
5–1	−59.861	−140.848	21.126	0.281
3–2	−11.560	−85.596	62.477	0.998
4–2	−76.118	−141.843	−10.393	0.013
5–2	−92.072	−165.948	−18.196	0.005
4–3	−64.558	−121.077	−8.038	0.015
5–3	−80.512	−146.333	−14.691	0.007
5–4	−15.954	−72.264	40.355	0.966

*Note:* The table shows the differences in mean hospital length of stay (LOS) across varying numbers of ERAS elements practiced. For example, implementing 5 ERAS elements compared to none reduces LoS by 128.3 h (*p* = 0.018).

Statistically significant reductions (*p* < 0.05) are observed when four or more elements are implemented.

### 3.9 Predictors of Extended Hospital in Bellwether Procedures

Age is significantly associated with prolonged stays (coefficient = 0.294, OR = 1.342, and *p* < 0.001). Female patients are less likely to experience extended stays compared to males (coefficient: −0.794, OR: 0.452, and *p* = 0.007). Nonscheduled procedures slightly decrease the odds of extended stays but lack statistical significance (coefficient: −0.107, OR: 0.898, and *p* = 0.073). The type of surgical procedure shows varying effects: CS tend to increase the odds of prolonged stays (coefficient: 0.479, OR: 1.615, and *p* = 0.059), whereas laparotomies do not show significant effects (coefficient: −0.289, OR: 0.749, and *p* = 0.223). Adherence to ERAS elements shows a progressive and significant reduction in prolonged stay likelihood, with adherence to two, three, four, and five elements each contributing to increasingly shorter stays (e.g., five elements: coefficient: −0.718, OR: 0.488, and *p* = 0.002), as further described in Table 6.

**TABLE 6:** Predictors of extended hospital length of stays (LOS) in Bellwether surgical procedures logistic regression summar ***y***.

Features		Coefficient	Odds ratio	Standard error	*p* value	95% CI lower	95% CI upper
Age	Age (Years)	0.294	1.342	0.111	< 0.001	1.213	1.875
Sex	Female	−0.794	0.452	0.249	0.007	0.310	0.826
	Male	—	—	—	—	—	—
Urgency of surgery	Nonscheduled	−0.107	0.898	0.522	0.073	0.943	7.465
	Scheduled	—	—	—	—	—	—
Surgical procedure type	Cesarean section (CS)	0.479	1.615	0.379	0.059	0.981	4.354
	Laparotomy	−0.289	0.749	0.350	0.223	0.326	1.291
	Open bone fracture (OBF)	—	—	—	—	—	—
Adherence to the ERAS elements	One element	−0.028	0.973	0.706	0.074	0.064	1.061
	Two elements	−0.330	0.719	0.696	0.02	0.045	0.723
	Three elements	−0.314	0.731	0.682	0.022	0.049	0.740
	Four elements	−0.873	0.418	0.669	0.002	0.029	0.416
	Five elements	−0.718	0.488	0.731	0.002	0.023	0.422
	No element	—	—	—	—	—	—

## 4 Discussion

The key finding of this study was that the adherence rate to selected ERAS guidelines recommendations in Ethiopian surgical facilities was 63.7%, and the ability to comply the five recommendations was associated with a reduction in LOS by 128 h for Bellwether surgical procedures. The two ERAS elements with the strongest association with a reduced LOS were the removal of the urinary catheter and early mobilization. PONV prophylaxis was not associated with a significant reduction in LOS. The significant reduction in LOS through adherence to five ERAS guidelines recommendations underscores the benefits of adherence to ERAS guidelines in low‐resource environments.

Although widely implemented in high‐income countries, with beneficial clinical outcomes, the implementation of ERAS guidelines in low‐resource settings offers unique challenges [1, 2, 3, 4, 5], even so, examples of successful ERAS programs in low‐resource settings exist [6, 7]. The results of this study found that most patients were classified as ASA 1 and ASA 2 (94.8%), indicating a relatively low‐risk patient population. Elements of ERAS guidelines are being implemented in Ethiopia with an adherence rate of 65.2% (1810). This highlights the potential for further improvement in adherence to ERAS guidelines in Ethiopia, which could lead to even greater reductions in LOS. However, it is important to consider the overall clinical picture and individual patient factors when determining the potential impact of ERAS guideline adherence on LOS.

The implementation of comprehensive ERAS guidelines is expected to reduce the LOS by 30%–50% [1]. This study examined five ERAS elements and found that their combined implementation significantly reduced the LOS, further supporting the potential benefits of adopting the full ERAS guidelines for improved patient outcomes. Length of stay (LOS) varied based on the type of surgery performed. For instance, although this study did not specify fracture locations, it highlighted that early mobilization significantly reduced LOS in patients treated for open fractures. In contrast, PONV prophylaxis did not demonstrate a measurable impact on LOS in these patients. Additionally, in the OBF group, there was no significant difference in LOS between patients with urinary catheters retained for more than 24 h and those without. These findings underscore the importance of tailoring ERAS guidelines to specific surgical disciplines and operations, even when applying simplified guidelines in a resource‐limited environment. In addition, this study confirms the importance of increasing compliance to all components of the bundle in reducing LOS, as a collective bundle is more important than individual components in improving quality metrics [18]. This has been shown in other studies [19].

Our study also found that administration of PONV prophylaxis was less commonly practiced in CS, compared to the expected 100% administration of PONV in this patient group, as suggested in the literature [20]. This result is consistent with a systematic review by Corso et al. [21], which showed low compliance with PONV prophylaxis in ERAS guidelines for obstetric cases. Further studies are essential to understand the reasons for this difference and explore potential strategies for improving the use of PONV prophylaxis in CS. Moreover, adherence to even one of the recommended ERAS elements reduced LOS by approximately 68 h compared to nonadherence, whereas full adherence significantly reduced LOS by 128 h. This is important for healthcare financing, especially as in this study, where 32 (5.8%) patients received the procedure for free, especially the CS group, with hospitals and government budgets covering expenses. Implementation of complete adherence to ERAS guidelines could lead to substantial hospital and government savings in low‐resource environments, consistent with other studies [22, 23]. Therefore, complete adherence is potentially linked to healthcare financing of these procedures in Ethiopia. Although CS‐specific ERAS guidelines exist [24, 25, 26], the current LMIC guidelines target abdominal and pelvic surgeries. Although the ERAS components evaluated in this study have been implemented for CS in Ethiopia, it is necessary to tailor these guidelines further and establish standardized ERAS protocols in obstetrics within LMICs. This is important in Africa, where women face a higher risk of severe postoperative complications following elective nonobstetric nongynecological surgery than international comparators [27].

To the best of our knowledge, this study is the first to assess ERAS practices across a large sample of various surgical procedures in Ethiopia and Africa, whereas previous studies have focused on specific procedures using smaller samples [28]. The availability of a perioperative registry data system in Ethiopia has facilitated this comprehensive analysis. These data are crucial for the ERAS implementation as they allow for the integration of more ERAS guidelines and recommendations into national perioperative practices and enable regular interactive audits [29, 30]. As we studied the most common surgeries (Bellwether surgical procedures) in Ethiopia, our findings can be extended to a wider trend of all other surgical procedures and perioperative care. The main finding is that following adherence to a specific and small set of ERAS recommendations in low‐resource settings could potentially reduce the LOS by about 5 days for Bellwether surgical procedures and adherence to early mobilization, early removal of urinary catheters, and early feeding each has shown a consistent reduction in LOS across all Bellwether surgical procedures. We acknowledge that variations in patient comorbidities, healthcare provider awareness, and resource availability may differ among the Bellwether procedures. These factors can influence how well ERAS recommendations are adopted and followed across the different procedures. Further research and improvements are needed, especially given the 35% nonadherence rate, for the limited assessment of individual patient characteristics and the evaluation of only five postoperative elements of the ERAS guidelines. This can be achieved by educating and training healthcare providers about the significance and benefits of adhering to ERAS guidelines as well as educating patients about the importance of following these recommendations to optimize their recovery and outcomes.

The limitations of this study include the retrospective nature of the data collection, which restricted our ability to evaluate specific patient and surgical characteristics not routinely documented in the registry, such as the distinction between upper and lower extremity fractures, associated injuries, type of bowel obstruction, and the comorbidities of patients across different surgical categories. Additionally, although adherence to the ERAS recommendations was evaluated based on the data recorded in the registry, we could not verify the accuracy and quality of the data entries due to the retrospective design of the study. The inability to ensure completeness in patient records may have also impacted the results. Future research could benefit from prospective data collection methods that capture these specific details to provide stronger evidence of the impact of ERAS guidelines on patient outcomes.

## Author Contributions

F.K.: conceptualization, project administration, investigation, methodology, analysis, writing – original draft. E.B.: data curation, analysis, investigation, visualization. T.K., B.D., B.M., E.P., R.O., S.M., B.B.: writing – review and editing, investigation. S.M., B.B.: supervision, conceptualization, methodology, resources. All authors approved the final manuscript.

## Ethics Statement

The study has been approved by the University of Cape Town Human Research Ethics Committee (HREC) REF 220/2024 and Arbaminch University with a protocol number of DG1445.

## Conflicts of Interest

The authors declare no conflicts of interest.

## Supporting information

Supporting Information S1
