# Supplementary material for: Adherence to Enhanced Recovery After Surgery (ERAS) With Bellwether Surgical Procedures in Ethiopia: A Retrospective Study
Source: World J Surg. 2025 Mar 20;49(4):1040–50. doi: 10.1002/wjs.12526 (PMC11994138; doi:10.1002/wjs.12526)

# Annex 1: Supplementary files

# Table 1: Types and Characteristics of Hospitals Included in the Study

| Type of hospital | Level of hospital | Number of hospital beds | Number of surgical beds | Average monthly surgical volume | Average monthly Obstetric procedures |
| --- | --- | --- | --- | --- | --- |
| Government | Tertiary | 511 | 190 | 1274 | 250 |
| Government | Tertiary | 500 | 108 | 287 | 35 |
| Government | Tertiary | 303 | 54 | 424 | 150 |
| Government | Secondary  (General) | 285 | 71 | 260 | 128 |
| Government | Tertiary | 171 | 34 | 261 | 109 |
| Government | Tertiary | 170 | 37 | 160 | 85 |
| Government | Tertiary | 80 | 35 | 139 | 66 |

**Figure 1: Missing data magnitude and pattern**


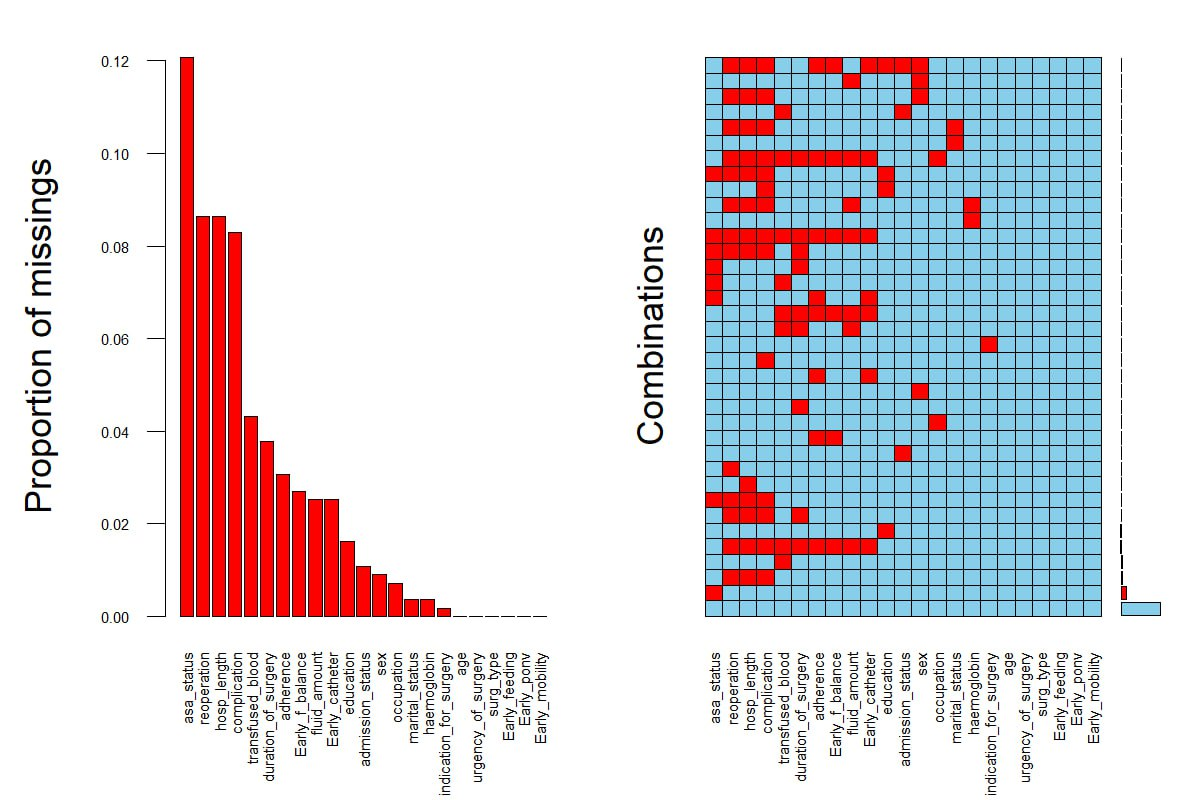

Supplement: Supplementary file 1 — Supporting Information S1 [file WJS-49-1040-s001.docx]
